# Cognitive Functions in Middle Aged Individuals Are Related to Metabolic Disturbances and Aerobic Capacity: A Cross-Sectional Study

**DOI:** 10.1371/journal.pone.0051132

**Published:** 2012-12-12

**Authors:** Maria Pedersen, Karin Kaereby Pedersen, Helle Bruunsgaard, Karen Suarez Krabbe, Carsten Thomsen, Kristine Færch, Bente Klarlund Pedersen, Erik Lykke Mortensen

**Affiliations:** 1 The Centre of Inflammation and Metabolism at Department of Infectious Disease, Rigshospitalet, The Faculty of Health Sciences, University of Copenhagen, Copenhagen, Denmark; 2 Department of Clinical Immunology, Rigshospitalet, University of Copenhagen, Copenhagen, Denmark; 3 Department of Radiology, Rigshospitalet, University of Copenhagen, Copenhagen, Denmark; 4 Steno Diabetes Center, Gentofte, Denmark; 5 Institute of Public Health and Center for Healthy Aging, University of Copenhagen, Copenhagen, Denmark; Mayo Clinic College of Medicine, United States of America

## Abstract

**Aims:**

Metabolic disturbances may contribute to cognitive dysfunction in patients with type 2 diabetes. We investigated the relation between cognitive impairment and metabolic deteriorations, low physical fitness, low-grade inflammation and abdominal obesity in middle aged individuals.

**Methods:**

We conducted a cross-sectional study including 40 to 65 year-old patients with type 2 diabetes and limited co morbidity (N = 56), age-matched individuals with impaired glucose tolerance (N = 56) as well as age-matched controls with normal glucose tolerance (N = 72). Specific cognitive functions were assessed with focus on verbal memory, processing speed, executive functions, and a composite overall mean score. Oral glucose tolerance test, VO_2_max test, systemic inflammation, DXA scanning and abdominal MRI were measured.

**Results:**

Multiple linear regression analyses adjusting for age, gender and verbal intelligence demonstrated that a low score in processing speed, executive functions and overall cognitive function were related to high fasting C-peptide, as well as low insulin sensitivity, beta-cell function and VO_2_max. Measurements of blood glucose, obesity and inflammation were not associated with cognitive function.

**Conclusion:**

Low cognitive scores are seen in middle aged individuals with hyperinsulinemia, low insulin sensitivity, beta-cell function and low aerobic capacity. These findings emphasize the importance of appropriate lifestyle and not only blood glucose control in prevention of cognitive disability.

## Introduction

Diabetes is associated with increased risk of cognitive dysfunction in elderly people [1]–[5]. The underlying mechanisms are multifactorial. However, in older individuals and in the severe states of type 2 diabetes, the presence of many confounding factors makes it difficult to determine the causative factors to cognitive disability. Thus, previous transient cerebral ischemia (TCI), stroke, myocardial infarction, or atherosclerosis are all strong predictors of cognitive dysfunction [6]. Associations between high HbA1c and cognitive dysfunction have been demonstrated in elderly [2] and middle aged [7] individuals. Also, in elderly patients with type 2 diabetes, acute deficits in working memory and attention were observed in the hyperglycemic state during a glucose clamp [8].

Dementia share many risk factors with type 2 diabetes such as physical inactivity [9], [10], inflammation [3] and obesity [11]–[13]. The hypothesized pathways linking dementia to type 2 diabetes are multiple including insulin resistance in brain [14], [15], decreased neuroplasticity [16] and cerebral atherosclerosis [10]. Also a sedentary lifestyle is associated with increased risk of dementia, and it has been observed that regular moderate exercise during midlife is related to higher cognitive performance in later life [17]. Physical inactivity also results in a chronic elevation of inflammatory biomarkers [18], which are also observed in patients with dementia and type 2 diabetes [19]. In addition, inflammatory mediators such as TNF-alpha are secreted from abdominal adipose tissue [11], and previous findings have shown that abdominal obesity to a higher degree than whole body obesity is associated with risk of cognitive decline in late life [20].

Cognitive function in later parts of life is substantially dependent on cognitive function and intelligence in young adulthood [21]. To take this into account, the present study incorporated an index of verbal intelligence, which is a measure of a cognitive function known to be preserved even after other cognitive functions have been impaired by brain damage or age related cognitive changes [22], [23].

The overall aim of the present cross-sectional study was to test the hypothesis that metabolic disturbances (low insulin sensitivity and chronic hyperinsulinemia) and related risk factors including low fitness level, low-grade inflammation and abdominal obesity are associated with cognitive functions in middle aged individuals.

## Research and Design Methods

In the present study 197 individuals aged 40 to 65 years were included. After initial screening as described below, 13 participants were excluded, resulting in a study sample of 184 individuals. To avoid severe states of type 2 diabetes and thereby high level of co- morbidity, patients treated with insulin were excluded. Other exclusion criterias were recent or ongoing infections, history of malignant cancer and severe chronic inflammatory diseases. Recruitment of participants was closely monitored to obtain three groups of participants with comparable age, gender and BMI, but with different glucose tolerance status: Normal glucose tolerance (NGT), impaired glucose tolerance (IGT) and type 2 diabetes. All participants had a clinical examination, electrocardiogram and screening blood tests including renal, hepatic and thyroid function, hemoglobin, white blood cell counts and electrolytes. To exclude conditions known to influence cognitive function, participants with a history of recurrent hypoglycemia, cerebral infections, epilepsy, traumatic brain injury, stroke, alcohol abuse, severe depression or impairment in reading, hearing, vision or motor skills were excluded from the cognitive test battery (n = 13), resulting in a study sample of 184 individuals. Eleven participants were treated for mild depression with either selective serotonin reuptake inhibitors or serotonin- and norepinephrine reuptake inhibitors (NGT, N = 4; IGT, N = 5 and type 2 diabetes, N = 2). None of the participants had any other known diseases suspected to affect neuropsychological functions.

Participants were primarily recruited from advertisements in local newspapers and three of the participants with impaired glucose tolerance were recruited from a register of known test subjects at Steno Diabetes Centre. Twenty-seven patients with type 2 diabetes received no medical treatment for diabetes, twenty-three patients were treated with metformin either as mono-therapy (n = 17) or in combination with a beta-cell stimulating drug (n = 6), and finally six patients had beta-cell stimulating drug as mono-therapy. To minimize the effect of different treatment regimes those with type 2 diabetes were not allowed taking any antidiabetic medication for one week preceding the OGTT.

### Ethics statement

All participants gave written informed consent before inclusion and the study was performed according to the Declaration of Helsinki and approved by The Regional Committee on Biomedical Research Ethics in Denmark (KF 01-141/04).

### Study protocol and procedures

Participants reported to the clinical department at two different study days. At the first study day participants arrived in the morning after an overnight fast. Blood samples, clinical examination and a neuropsychological assessment were performed; the latter after a common Danish breakfast consisting of oatmeal and muesli. Withdrawal criteria were blood pressure >180/110 mmHg, fasting glucose concentrations >12 mmol/l and HbA1c >11%.

On a separate study day (within 8 to 21 days after first visit) an oral glucose tolerance test (OGTT) was performed. Participants reported to the clinical department after an overnight fast.

### Neuropsychological assessment

The neuropsychological tests were selected to assess cognitive functions which may be affected in middle-aged individuals with type 2 diabetes [24].The tests represent memory, processing speed, attention and executive function. The neuropsychological assessments were administered at visit 1, before results regarding physical fitness, abdominal obesity, as well as the results from the OGTT were available. This was done to avoid the tester to be biased by those results. All tests were administered by the same medical doctor (first author) trained in administering the included tests. Individual test scores were reported as raw-scores and to enable calculation of composite scores the raw scores were transformed to standardized z-scores based on the mean and the standard deviation of the entire study sample.

To investigate the verbal intelligence we used two different tests: 1) The Danish Adult Reading Test (DART) is a Danish version of the English New Adult Reading Test (NART) [25], [26]. The participant was required to read 50 words and the pronunciations were evaluated as either correct or incorrect by the tester. 2) The Danish version of the Information subtest of Weschlers Adult Intelligence Scale-III (WAIS-III) [27], [28] where participants were asked 28 questions on general knowledge. To derive a composite index of verbal intelligence the mean of the two z-scores was calculated.

A modified Rey Auditory Verbal Learning Test (RAVLT) was administered to assess verbal learning and memory. Participants were read a list of 15 unrelated words and were asked to repeat the words after each trial. The total number of correct words in three trials was summarized [27], [29].

Processing speed was assessed with two different tests. Symbol Digit Modalities Test (SDMT), requires the participant to write as many pairs of symbols and digits as possible in 90 seconds. In addition, Trail Making Part A [30] was administered to test processing speed. Trail Making Part A consists of 25 numbers, which must be connected in arithmetic order. Both tests presumably reflects speed of information and motor ability [27] and a composite score “Processing Speed” were calculated. Executive functions were assessed with Trail Making part B, consisting of numbers and letters, which must be connected by alternating 1-A-2-B etc. [30]. Additionally, in a category fluency task, the participants were asked to generate as many animals as possible within 60 seconds and in the letter fluency task, the participants were asked to generate as many words as possible starting with the letter “S” within 60 seconds [27]. A composite score of “Executive functions” was derived as the mean of Trail Making B and the two fluency scores. Regarding trail making A and B, high raw score was equivalent to a low z-score and thereby low cognitive performance. Finally, a composite overall mean cognitive score was derived from the mean z-scores from the six individual tests mentioned above.

All participants were asked to complete the Major Depression Inventory Questionnaire [31] as well as to report the number of years of education.

### Assessment of glucose metabolism

A standard 75 gram oral glucose tolerance test was performed. After an overnight fast, the participants consumed 75 g glucose dissolved in 350 ml room tempered water within five minutes. Blood samples for measurement of plasma glucose, serum insulin and serum C-peptide were collected at −30, 0, 30, 60, 90 and 120 min relative to the time of glucose ingestion. Plasma and serum levels of glucose, insulin, and C-peptide were sent for immediate analysis at the hospital laboratory (colorimetric hexokinase assay used for plasma-glucose, and ELISA for serum C-peptide and -insulin). Participants were categorized as NGT, IGT or type 2 diabetes based on fasting and 2 hrs plasma glucose concentration according to the WHO criteria [32]. A dynamic index of insulin sensitivity was calculated as Matsuda composite index based on five time points [33]; a high Matsuda composite index is equivalent to a high insulin sensitivity. To obtain a measure of steady state glucose metabolism, pancreatic beta-cell function was calculated by using the homeostatic model assessment (HOMA-B) [34]. A dynamic index of beta-cell function, the insulinogenic index was calculated as the change in insulin concentration (ΔI) divided by the change in glucose concentration (ΔG) from 0 to 30 min during the OGTT [35].

### Physical fitness and body composition

Physical fitness was assessed by a single-stage sub-maximal model (Aastrand test) [36]. This sub-maximal test is feasible in individuals not used to intense physical activity [37]. Participants were told to exercise for six minutes on an ergometer bike (Monark 839E, Monark Ltd, Varberg, Sweden) with a workload equivalent to a heart rate between 110 and 150 bpm. Based on gender, age, body weight, workload and heart rate an estimate of VO_2_max (L/min and ml/min/kg) was calculated.

Dual-energy X-ray absorptiometry (DXA) whole body scanner (GE Medical Systems Lunar Prodigy Advance, Fairfield Connecticut, USA) was used to assess body composition including whole body fat- and fat-free mass. Intra abdominal fat content was assessed by 3-Tesla MRI (Siemens Magnetom Total imaging matrix magnetic resonance scanner, Erlangen, Germany). Sixty slides T1- weighted abdominal slides were acquired during breath hold with a slide thickness of 5 mm with no overlap. Intra abdominal fat was analyzed with multi-slide model using MANGO (Multi-Image Analysis GUI) version 2.5 developed at the Research Imaging Center (The University of Texas Health Science Center, San, Antonio, TX). Any adipose tissue located from diaphragm to first sacral vertebra, except subcutaneous tissue, was characterised as intra abdominal fat.

### Assessment of Inflammatory biomarker

Analysis of plasma interleukin-6 (IL-6) and Tumor Necrosis Factor-Alpha (TNF-α) were measured by MSD (Meso Scale Discovery, Gaithersburg USA), samples were run in duplicates and mean concentrations were calculated. In order to control intra assay variability three control samples were run on all MSD-plates, CV<25% for IL-6 and <15% for TNF-α were achieved.

### Statistical analyses and calculations

Normality of data was tested by Kolmogorov-Smirnov. If data were not normally distributed a logarithmical transformation was performed (Trail Making A and B, fasting insulin and glucose, HOMA-B, insulinogenic index, Matsuda composite index, VO2max, IL-6 and TNF-α). Significance of differences between the three metabolic groups was tested using ANOVA, followed by Tukeys post hoc test and adjustment for age and gender were conducted for RAVLT, SDMT, Trail making tests and Word mobilization tests. Distribution of gender was tested by a Chi-square test. Linear regression analyses were performed to identify relationships between composite cognitive scores (dependent variables) and metabolic parameters, physical fitness, body composition as well as inflammation. All analyses were performed with adjustment for age, gender and verbal intelligence. No adjustment for multiple testing was done as all tests were predefined.

Parameters found to be related to cognitive function (fasting c-peptide, Matsuda composite index, insulinogenic index and VO_2_max), quartiles were calculated and a risk-score between zero and three for each of the parameters were summed. Three groups were constructed based on cumulative risk score. Low risk includes participants with low fasting c-peptide, high beta-cell function, high insulin sensitivity and aerobic capacity (cumulative risk score = 0–4). High risk includes participants with high fasting c-peptide, low beta-cell function, insulin sensitivity and aerobic capacity (cumulative risk score = 8–12). Differences in cognitive scores between groups are tested with one-way ANOVA and tukey post-hoc. As model control, normality of residuals and a plot of residuals against predicted values were performed. All analyses were performed using SAS 9.1 (SAS Institute, Cary, NC, USA).

## Results

Clinical characteristics of the study group are presented in Table 1. Patients with type 2 diabetes had higher HbA1c, fasting insulin, -C-peptide and -glucose compared to participants having NGT. The Matsuda composite index of insulin sensitivity were lower in participants having IGT and type 2 diabetes compared to NGT, whereas we found a compensatory higher beta-cell function (HOMA-B and insulinogenic index) in the individuals with impaired glucose tolerance compared to type 2 diabetes. Regarding inflammatory biomarkers (IL-6 and TNF-α), type 2 diabetes patients had higher levels compared with NGT subjects. Across the groups there was no statistically significant difference in the index of verbal intelligence (DART and Information), years of education or depression-score. Furthermore, we were not able to demonstrate significant group differences according to glucose tolerance status on any of the individual cognitive test scores (Table 2).

**Table 1:** Characteristics of the study population.

	Normal glucose tolerance, (n = 72)	Impaired glucose tolerance, (n = 56)	Type 2 diabetes (n = 56)	P-value
Age (years)	53 (48–60)	53 (47–60)	58 (51–62)	0.06
Male (%)^a^	46	46	54	0.33
**Metabolic data**				
HbA1c (%)	5.5 (5.3–5.7)	5.7 (5.5–6.0)*	6.6 (6.0–7.2) *	<0.01
Glucose, fasting (mmol/l)	5.2 (4.8–5.7)	5.5 (5.2–6.1)	7.3 (6.1–8.9) *	<0.01
Insulin, fasting (pmol/l)	41 (22–56)	52 (36–86)*	57 (29–100) *	<0.01
C-peptide, fasting (nmol/l)	0.70 (0.53–0.87)	0.89 (0.70–1.18) *	1.00 (0.69–1.23) *	<0.01
Matsuda composite index	5.8 (3.7–10.4)	3.8 (2.4–5.9) *	3.4 (2.2–5.5) *	<0.01
HOMA B	50.6 (29.2–86.7)	59.7 (39.7–89.8)	29.5 (16.1–70.9) *	<0.01
Insulinogenic index (pmol/mmol)	85.7 (51.0–124.4)	70.9 (44.1–102.3)	23.3 (12.5–42.1) *	<0.01
**Fitness**				
VO_2_ max (L/min)	2.3 (1.9–3.0)	2.3 (2.0–2.7)	2.0 (1.6–2.6) *	0.02
VO2max/kg (ml/min/kg)	25.8 (20.2–34.4)	25.0 (20.5–29.2)	24.1 (19.1–26.4) *	0.04
**Inflammatory markers**				
TNF-alpha (pg/ml)	2.4 (2.0–2.8)	2.7 (2.4–3.1)	2.7 (2.2–3.5) *	0.02
IL-6 (pg/ml)	1.5 (1.1–2.4)	2.0 (1.5–3.3)	2.4 (1.4–4.1) *	0.02
**Body composition**				
BMI (kg/m^2^)	29.7 (24.5–35.3)	32.2 (28.2–37.1) *	28.7 (25.9–33.0)	<0.01
Intra abdominal fat content (l)	3.1 (1.5–4.7)	4.2 (3.3–6.4) *	3.9 (2.5–4.8)	<0.01
Whole body fat percent	34.6 (26.6–41.9)	36.6 (32.4–44.0)	34.1 (25.8–41.3)	0.04

Data are expressed as median and 25% and 75% quartiles. HbA1c, glycated hemoglobin; HOMA B, beta-cell function as measured by homeostatic model assessment [34]. P-values refer to parametric analysis of variance. (a) data are tested by Chi-square test.

* denotes significant different (P<0.05) from individuals with normal glucose tolerance, corrected with Dunnett's test.

**Table 2:** Cognitive function and depression score.

	Normal glucose tolerance	Impaired glucose tolerance	Type 2 diabetes	P-value
DART (correct pronunciations)	38 (30–42)	36 (31–39)	36 (29–43)	0.76
Information (correct answers)	20 (16–23)	18 (15–22)	18 (15–22)	0.21
Major Depression Inventory score	3 (2–6)	3 (1–6)	4 (1–7)	0.15
Years of education (years)	14 (13–16)	13 (12–14)	13 (12–16)	0.10
RAVLT (no of word)	22 (18–24)	22 (19–25)	20 (18–23)	0.33
SDMT (correct pairs)	51 (46–57)	50 (47–57)	50 (43–54)	0.50
Trail Making A (sec)	30 (23–37)	30 (24–37)	31 (25–40)	0.69
Trail Making B (sec)	69 (57–85)	70 (53–81)	72 (63–98)	0.13
Word mobilization, animal (word)	26 (22–30)	26 (23–30)	24 (21–27)	0.36
Word mobilization, letter (word)	15 (11–18)	15 (12–19)	15 (13–17)	0.51

Data are expressed as median and 25% and 75% quartiles. Parametric statistics are performed and adjusted by age and gender for RAVLT, SDMT, Trail making tests and Word mobilization tests. DART, The Danish Adult Reading Test; Information, Danish version of the Information subtest of Weschlers Adult Intelligence Scale-III; RAVLT, Rey Auditory Verbal Learning Test and SDMT, Symbol Digit Modalities Test. High Trail Making A and B value are considered as low cognitive score.

### Cognitive function and measurements of metabolic disturbances, fitness level, inflammation and central obesity

Results from multiple linear regression models testing explanatory metabolic variables on specific cognitive skills are presented in Tables 3. Low scores in processing speed (SDMT and Trail Making A) were associated with high fasting C-peptide, low insulin sensitivity and low fitness level. Low scores in executive functions (Trail Making B and word mobilization) were associated with high levels of C-peptide, decreased beta cell function and low fitness level. Analysis of the composite overall cognitive mean showed that low composite scores were associated with high fasting insulin, low beta-cell function, insulin sensitivity and fitness level.

**Table 3:** Multiple linear regressions between cognitive functions and parameters of glucose metabolism, fitness, body composition and inflammation.

	Memory		Processing speed		Executive functions		Overall cognitive scores	
	Standardized β	P-value	Standardized β	P-value	Standardized β	P-value	Standardized β	P-value
Fasting glucose^a^	−.06	.39	−.04	.57	−.07	.29	−.07	.30
HbA1c^a^	−.01	.85	−.07	.29	−.07	.29	−.07	.27
C-peptide, fasting	−.09	.21	−.21	<.01	−.14	.04	−.18	<.01
Insulinogenic a	.05	.50	.10	.12	.17	.01	.14	.02
Matsuda-index^a^	.10	.15	.17	.01	.08	.24	.13	.04
VO2 max (L/min)^a^	.11	.18	.22	<.01	.19	.01	.22	<.01
BMI (kg/m^2^)	.09	.19	.06	.34	.09	.21	.09	.15
Intraabdominal fat (kg)	.12	.18	.01	.86	.08	.38	0.7	.36
Interleukin-6^a^	−.002	.97	−.11	.10	−.06	.38	−.08	.23
Tumor necrosis factor-alpha^a^	−.07	.29	−.04	.58	−.01	.93	−.03	.59

a Statistical analysis are performed on logarithmic transformed data. Model is adjusted by age, gender and verbal intelligence. A low z-score are considered as low cognitive score. Memory score is measures of RAVLT; processing speed is composite score of SDMT and Trail making A; Executive functions is composite score of Trail making B and word mobilizations tests; and composite overall cognitive score is a mean of all cognitive test scores.

No clear relations of the cognitive scores with either inflammation or body composition were demonstrated.

### Cognitive function in relation to composite risk scores

**Figure 1:**
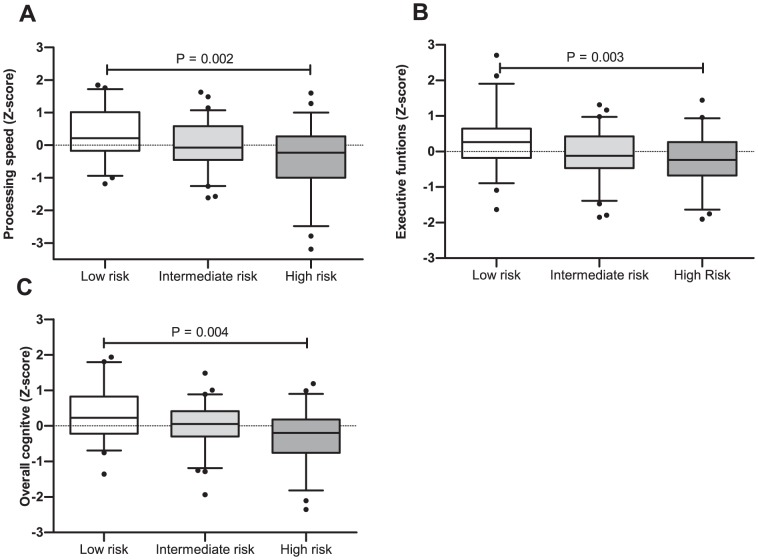
Box and whiskers plot of cognitive Z- scores in relation to “cumulative risk-score”. Z-scores of processing speed, executive function and overall cognitive score in three different risk groups. Box and whiskers plot presents quartiles and 5–95 percentiles. Differences between groups are tested with one-way ANOVA and tukey post-hoc. Processing speed (A), executive functions (B) and overall cognitive score (C) were significantly lower in individuals allocated to high risk group compared to low risk. Low risk includes participants with low fasting c-peptide, high beta-cell function, high insulin sensitivity and aerobic capacity (cumulative risk score = 0–4). High risk includes participants with high fasting c-peptide, low beta-cell function, insulin sensitivity and aerobic capacity (cumulative risk score = 8–12).

Based on a cumulative risk score from fasting C-peptide level, Matsuda composite index, beta-cell function and physical fitness level, three risk groups were constructed; Low risk (n = 57); Intermediate risk (n = 72) and high risk (n = 55), Table 4. Figure 1 demonstrates composite z-scores for processing speed, executive function and the composite overall mean. For the three composite cognitive scores, participants allocated to “high risk” had significantly lower z-scores compared to participants allocated to “low risk”.

**Table 4:** Quartiles of variables related to cognitive function.

Variable	High risk,	High to intermediate risk	Intermediate to low risk	Low risk
	Score = 3	Score = 2	Score = 1	Score = 0
C-Peptide, fasting (nmol/l)	>1.1	0.8–1.1	0.6–0.8	<0.6
Insulinogenic index	<29.7	29.7–57.7	57.7–100.1	>100.1
Matsuda composite index	<2.7	2.7–4.5	4.5–6.9	>6.9
VO2max (L/min)	<1.8	1.8–2.3	2.3–2.7	>2.7

For each variable related to cognitive function, quartiles and risk score is calculated and summarized to a “cumulative risk score” for each individual (between zero and twelve).

## Conclusions

The novel findings of this study were that low cognitive function is related to impaired glucose metabolism as well as low fitness level in middle aged individuals presenting a broad continuum of metabolic disturbances, physical fitness level and body weight.

To evaluate cognitive function in relation to risk factors associated with type 2 diabetes, we implemented a multivariate model adjusted for age, gender and verbal intelligence. In middle aged individuals, verbal intelligence can be assumed to be preserved even if other specific cognitive functions have declined and consequently our index of verbal intelligence provides an indirect method for adjusting for individual differences in peak prior cognitive function in young adulthood. When we adjust for individual differences in verbal intelligence and to the extent the test of the specific cognitive functions are related to verbal intelligence, this model reflects decline in cognitive function.

Processing speed (SDMT and Trail Making A) and executive functions (Trail Making B and word mobilization) were inversely related to levels of fasting C-peptide. In accordance with our data, Choleton and coworkers [15] concluded in a recent review that long lasting hyperinsulinemia and insulin resistance, as is the case in the major part of our study-population, are followed by a downregulation of insulin receptors on the blood-brain barrier and reduced insulin transport into the brain. In addition, cerebral administration of insulin with euglycemia improves cognitive function with special effect on memory function in humans [38], [39].

In the present study population, covering a broad spectrum of insulin secretion and action, we did not observe any difference in cognitive functions between the glucose tolerance groups. This may be explained by the complexity of the pathogenesis of type 2 diabetes and the overlap regarding insulin sensitivity and beta-cell function. Our data demonstrate that neither fasting glucose nor HbA1c or glucose tolerance as such are risk factors for minor cognitive dysfunction. Instead, the very early stages of glucose dysregulation, such as low insulin sensitivity and compensatory chronic hyperinsulinemia seem to play a role. Thus, insulin sensitivity and beta-cell function per se, and not diagnostic group, affects cognitive function. Therefore, screening for type 2 diabetes using HbA1c, fasting or 2-hour post OGTT glucose concentrations does not catch individuals with chronic hyperinsulinemia and increased risk of cognitive dysfunction.

The finding that a high fitness level was related to high processing speed and increased executive functions, is consistent with other data, showing a beneficial effect of physical activity on cognitive function in elderly individuals [9], [40]. The effects of exercise may influence many pathways. It has been suggested that contracting muscles secrete so-called myokines that mediate an anti-inflammatory effect [18]. Exercise also increases peripheral insulin sensitivity, which may be related to higher insulin level in brain [15]. In addition, exercise is suggested to improve neuroplasticity [16], as well as cardiovascular and cerebrovascular function [10].

In contrast to our hypothesis, we were unable to show an association of cognitive dysfunction to inflammation or intra abdominal fat, which contrasts previous findings [13]. The divergence may be explained by the fact that we included a younger and healthier study population. Thus, it is possible that a relationship between inflammation, central obesity and cognitive function are driven by many years of chronic inflammation originating from the inflamed visceral fat.

One major limitation of this study is the cross-sectional design, which only allows us to demonstrate associations. In theory, it is possible that low cognitive function causes lower physical activity and therefore leads to metabolic deteriorations. On the other hand, the literature supports our findings as mentioned above. To verify our results, causality needs to be further investigated in large-scale prospective studies with focus on subtle changes in glucose regulation and/or fitness level. The strengths of the present study are the well-characterized study-population, with age and BMI- matched groups, the dynamic estimation of beta-cell functions and insulin sensitivity from an OGTT as well as body composition from DXA-scans and MRI. Furthermore, by including an index of verbal intelligence as a covariate, it was possible to analyze effects on cognition independent of young adult individual differences in cognition.

In conclusion, our results demonstrate an association between low cognitive function and metabolic deterioration as well as low fitness level. The results thereby emphasize the importance of an active lifestyle to prevent cognitive dysfunctions in middle-aged humans.
